# HMGB1 promotes hair growth via the modulation of prostaglandin metabolism

**DOI:** 10.1038/s41598-019-43242-2

**Published:** 2019-04-30

**Authors:** Ji-Hye Hwang, Howard Chu, Yuri Ahn, Jino Kim, Do-Young Kim

**Affiliations:** 1 Department of Dermatology and Cutaneous Biology Research Institute, Yonsei University College of Medicine, Seoul, Korea; 2 New Hair Institute, Seoul, Korea

**Keywords:** Skin diseases, Translational research

## Abstract

Unexpected hair growth can occur after tissue injury. The pathogenic mechanism for this phenomenon is unknown but is likely related to inflammatory mediators. One such mediator is high-mobility group box 1 (HMGB1), a ubiquitous nuclear protein that is released from cell nuclei after tissue damage. To elucidate the effect of HMGB1 on hair growth and understand its mechanism of action, we evaluated the effect of HMGB1 treatment on hair shaft elongation and on mRNA and protein expression in cultured human dermal papilla cells (hDPCs). HMGB1 enhanced hair shaft elongation in an *ex vivo* hair organ culture. In hDPCs, HMGB1 treatment significantly increased mRNA and protein expression levels of prostagladin E synthases. HMGB1 also stimulated prostaglandin E2 (PGE_2_) secretion from hDPCs. Finally, blocking the receptor for advanced glycation end-products, a canonical HMGB1 receptor, inhibited HMGB1-induced PGE_2_ production and hair shaft elongation. Our results suggest that HMGB1 promotes hair growth via PGE_2_ secretion from hDPCs. This mechanism can explain the paradoxical phenomenon of trauma-induced hair growth. Thus, HGMB1 can be a viable therapeutic target for the treatment of alopecia.

## Introduction

Tissue injury can incidentally induce unexpected hair growth. For example, laser-induced paradoxical hypertrichosis occurs in 0.01~1.9% of patients treated with laser epilation^1^. Head and neck surgery, ultraviolet radiation exposure, and viral infection are also associated with hypertrichosis^2–4^. This phenomenon is likely related to inflammatory mediators and sub-therapeutic thermal injury causing induction of the hair cycle^5^; however, the exact cause is unknown. Overall, this mechanism of post-trauma or post-inflammatory hypertrichosis likely involves multiple biochemical mediators^4^.

One class of potential mediators is damage-associated molecular patterns (DAMPs). DAMPS are intracellular molecules released by injured tissues^6^. A canonical DAMP is high-mobility group box 1 (HMGB1), a ubiquitous nuclear non-histone protein. This small nuclear protein is involved in DNA transcription, replication, repair, and compaction^7^. Under conditions of stress or damage, HMGB1 is released actively or passively from various cells and acts as a critical factor in acute tissue injury^8,9^.

HMGB1 has also been implicated in regeneration processes. Under the influence of HMGB1, stem cells move toward an area of inflammation, contributing to tissue regeneration^10^. Furthermore, HMGB1 inhibition delays wound healing in normal mice, confirming its importance to tissue repair^11^. However, whether HMGB1 and its signalling partners play a role in hair growth is unknown. Because it is released upon injury, HMGB1 may have a role in post-traumatic or post-inflammatory hypertrichosis. In this study, we evaluated the effect of HMGB1 on hair shaft elongation in an *ex vivo* hair organ culture model and investigated the possible mechanisms of HMGB1-induced hair growth.

## Results

### HMGB1 stimulates hair shaft elongation in human hair follicles

Treatment with 25 to 200 ng/ml of HMGB1 enhanced hDPC proliferation *in vitro* without toxicity in a dose-dependent manner (p < 0.05; Supplementary Fig. 1). However, HMGB1 concentrations above 500 ng/ml were cytotoxic (data not shown). Accordingly, an HMGB1 concentration of 200 ng/mL was used for subsequent experiments.

**Figure 1:**
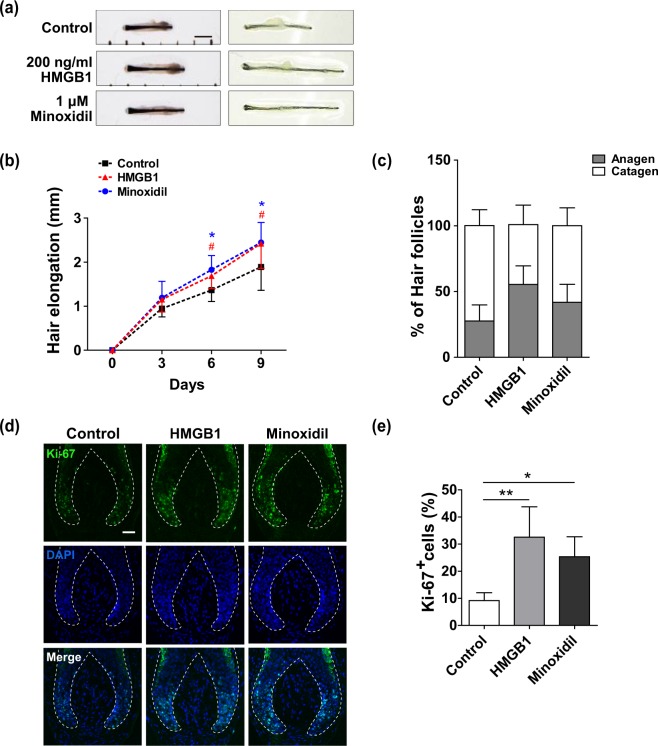
HMGB1 stimulates hair shaft elongation in human hair follicles. Isolated human hair follicles were treated with 200 ng/ml HMGB1 or 1 μM minoxidil for 9 days. (**a**) The photo panels show representative hair follicles from each group at day 0 (left) and 9 (right). Scale bar = 1 mm (**b**) Quantification of hair shaft growth rates. Changes in hair shaft length were measured using a stereomicroscope at 3, 6, and 9 days after culturing the cells. Data are shown as the mean ± SD of five independent donors (at least 90 follicles in each experiment). p-values were determined by ANOVA followed by Bonferroni post-hoc test. # p < 0.05, comparison between HMGB1 vs. control; *p < 0.05, comparison between minoxidil vs. control. (**c**) The percentage of hair follicles in each cycle stage at 9 days after treatment with 200 ng/ml HMGB1 or 1 μM minoxidil was assessed by morphological analysis. Data are shown as mean ± SD. (**d**) Immunofluorescence staining for Ki-67 (green) was performed after incubation of hair follicles with 200 ng/ml HMGB1 or 1 μM minoxidil for 3 days. 4′-6-diamidino-2-phenylindole (DAPI; blue) was used to counterstain the nuclei. Scale bar = 50 μm. (**e**) Ki-67^+^ positive cells in the matrix region were quantified. The results are shown as mean ± SD of three independent experiments. *p < 0.05 and **p < 0.01 compared with control group using one-way ANOVA.

To assess the role of HMGB1 on anagen hair follicles, lower anagen hair follicles micro-dissected from healthy human scalps were cultured with 200 ng/mL recombinant human HMGB1 or 1 μM minoxidil for 9 days. Changes in hair shaft lengths over time were measured. This revealed that HMGB1 treatment significantly increased hair elongation (p < 0.001; Fig. 1a,b) relative to untreated control follicles. This HMGB1-induced enhancement was comparable with 1 μM minoxidil, a representative positive control. Importantly, this effect became significant for both the HMBG1 and minoxidil treated groups beginning at 6 days. We also assessed the percentage of anagen and catagen hairs, which revealed that 56% of hair follicles remained in anagen after 9 days when cultured with HMGB1 relative to only 33% for untreated controls (Fig. 1c).

We next wanted to determine the effect of HMGB1 on hair follicle proliferation. Immunofluorescence analysis using Ki-67 as a marker for proliferation (Fig. 1d) revealed that HMGB1 treatment for 3 days significantly increased the hair follicle proliferation rate (32.5%) compared with untreated controls (9.1%; p < 0.01). This effect was also comparable to that of minoxidil (Fig. 1e). These findings suggest that HMGB1 is a novel hair growth-promoting mediator.

### HMGB1 increases PGE_2_ synthases and PGE_2_ production in hDPCs

**Figure 2:**
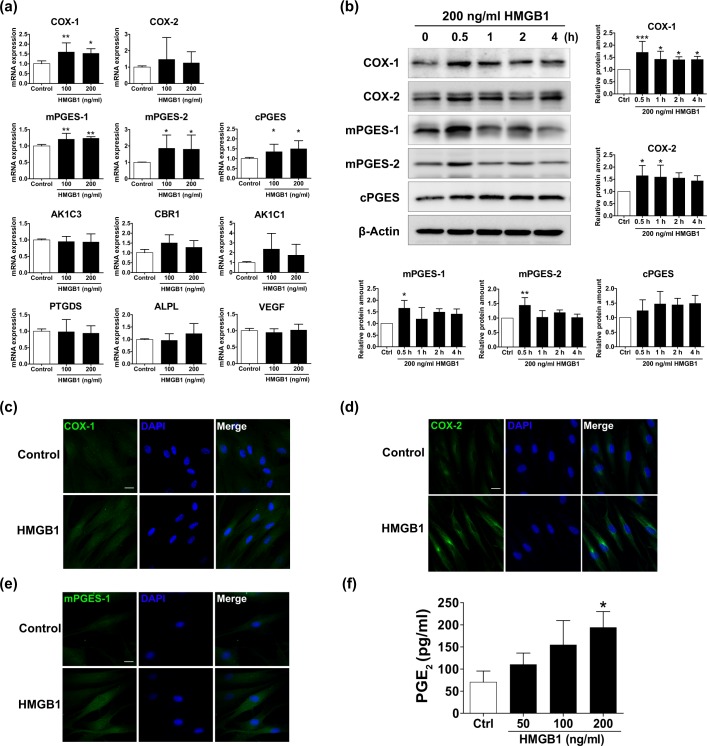
HMGB1 increases prostaglandin E synthase transcription in human dermal papilla cells (hDPCs). (**a**) mRNA expression of cyclooxygenase-1 (COX-1), cyclooxygenase-2 (COX-2), microsomal prostaglandin E synthase-1 (mPGES-1), microsomal prostaglandin E synthase-2 (mPGES-2), cytosolic prostaglandin E (cPGES), aldo-keto reductase family 1 member C1 (AKR1C1), aldo-keto reductase family 1 member C3 (AKR1C3), carbonyl reductase-1 (CBR-1), prostaglandin D synthase (PTGDS), alkaline phosphatase (ALPL), and vascular endothelial growth factor (VEGF) was examined in hDPCs treated with 100 or 200 ng/ml HMGB1 by real-time PCR analysis. The relative mRNA levels were normalized to *GAPDH*. The results are expressed as mean ± SD of three independent experiments. *p < 0.05 and **p < 0.01 compared to control group using ANOVA. (**b**) hDPCs were treated with 200 ng/ml HMGB1 for different incubation periods (0.5, 1, 2, and 4 h). The protein levels of COX-1, COX-2, mPGES-1, mPGES-2, and cPGES were measured using western blot analysis. β-actin served as a loading control. The results are expressed as mean ± SD of three independent experiments. *p < 0.05, **p < 0.01, and ***p < 0.001 compared with control group using one-way ANOVA followed by Bonferroni’s test. Representative images of immunofluorescence staining for (**c**) COX-1, (**d**) COX-2, and (**e**) mPGES-1 in hDPCs cultured with 200 ng/ml HMGB1 for 30 min. 4′-6-diamidino-2-phenylindole (DAPI; blue) was used to counterstain the nuclei. Data are representative of three independent experiments. Scale bar = 20 μm. (**f**) PGE_2_ secretion from hDPCs was determined by ELISA following treatment with various concentrations of HMGB1 (50, 100, and 200 ng/ml) for 4 h. The results are expressed as mean ± SD of three independent experiments. *p < 0.05 compared with control group using one-way ANOVA.

Dermal papilla, an aggregate of specialized fibroblasts located at the proximal end of a hair follicle, plays key roles in regulation of follicular development and hair cycle and supplies inductive signals required for proliferations of matrix keratinocytes^12^. To investigate possible signalling pathways involved in HMGB1-induced hair shaft elongation, we first used a growth factor array to screen the secretome of hDPCs. HMGB1 did not significantly increase the production of any growth factors screened (Supplementary Fig. 2). Because prostaglandins have recently been recognized as key growth regulating factors^13^, we next performed quantitative RT-PCR to quantify the mRNA levels of prostaglandin synthases in hair bulbs. This revealed that HMGB1 treatment substantially upregulated mRNA expression of PGE_2_ synthases, mPGES-1, mPGES-2, and cPGES in hDPCs (Fig. 2a). Specifically, we observed a 1.2-fold increase in *mPGES-1* expression, a 2-fold increase in *mPGES-2*, and a 1.5-fold increase in *cPGES* relative to control treatment. No significant changes were observed in the mRNA levels of PGF_2a_ synthases, AKRIC1, AKRIC3, and CBR1, and PGD_2_ synthase PTGDS when treated with HMGB1. Hence, further experiments were focused on PGE_2_ and HMGB1.

To verify the changes we observed in mRNA expression, we next measured the protein levels of PGE_2_ synthases by western blot. The experiments were performed in accordance with the finding of early induction of COX in previous studies^14,15^. This revealed that HMGB1 treatment significantly increased protein expression of COX-1, COX-2, mPGES-1, and mPGES-2 in hDPCs by approximately 1.5-fold relative to the control (Fig. 2b). Importantly, these increases occurred within 0.5 h of HMGB1 treatment. These results were visualized and confirmed using immunofluorescent staining (Fig. 2c–e).

**Figure 3:**
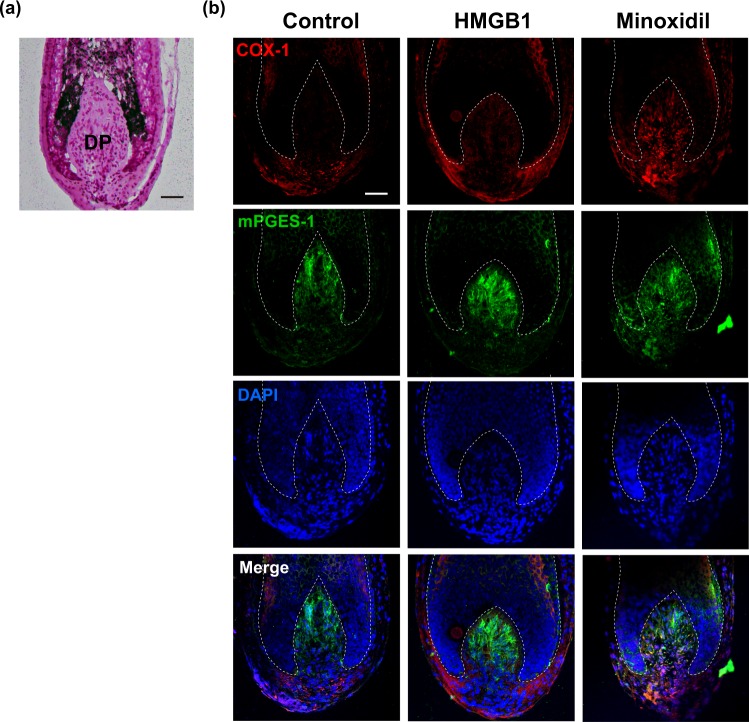
HMGB1 increases prostaglandin E synthase expression in human hair follicles. (**a**) Hematoxylin and eosin staining on the lower part of a cultured hair follicle. DP, dermal papilla. (**b**) Immunofluorescence staining for COX-1 (red) and mPGES-1 (green) in human hair follicles after 3 days of treatment with 200 ng/ml HMGB1 or 1 μM Minoxidil. 4′-6-diamidino-2-phenylindole (DAPI; blue) was used to counterstain the nuclei. Data are representative of ~4–5 hair follicles per each condition from three independent experiments. Scale bar = 50 μm.

Furthermore, ELISA analysis confirmed that HMGB1 treatment significantly elevated PGE_2_ secretion from hDPCs at 4 hours post-treatment (Supplementary Fig. 3). We also observed that this effect of HMGB1 on PGE_2_ production was in a dose-dependent manner (Fig. 2f). In order to verify the different effects of HMGB1 depending on its chemical forms, we tested subunits of HMGB1 and a form with different redox status. Both box A and B did not induce PGE_2_ production (Supplementary Fig. 4a). Pre-treatment of 5 mM dithiothreitol (DTT) can reduce a disulfide form of HMGB1 which we used (R&D Systems, #1690-HMB-050, Supplementary Fig. 4b). Interestingly, both redox forms of HMGB1 similarly increased PGE_2_ production (Supplementary Fig. 4c). Collectively, subunit of HMGB1 alone is not sufficient to induced PGE_2_ production but either redox forms of HMGB1 can stimulate PGE_2_ production in hDPCs. To explore biological relevance, we tested HMGB1 in organ culture model. HMGB1 increased expressions of COX-1 and mPGES-1, which was confirmed by confocal microscopy in isolated human hair follicle after treating with 200 ng/ml HMGB1 for three days. Immunofluorescent analysis also revealed that HMGB1 or minoxidil treatment both increased COX-1 and mPGES-1 expression in hair follicles compared to control levels (Fig. 3).

### RAGE is the target receptor for HMGB1-induced PGE_2_ production

**Figure 4:**
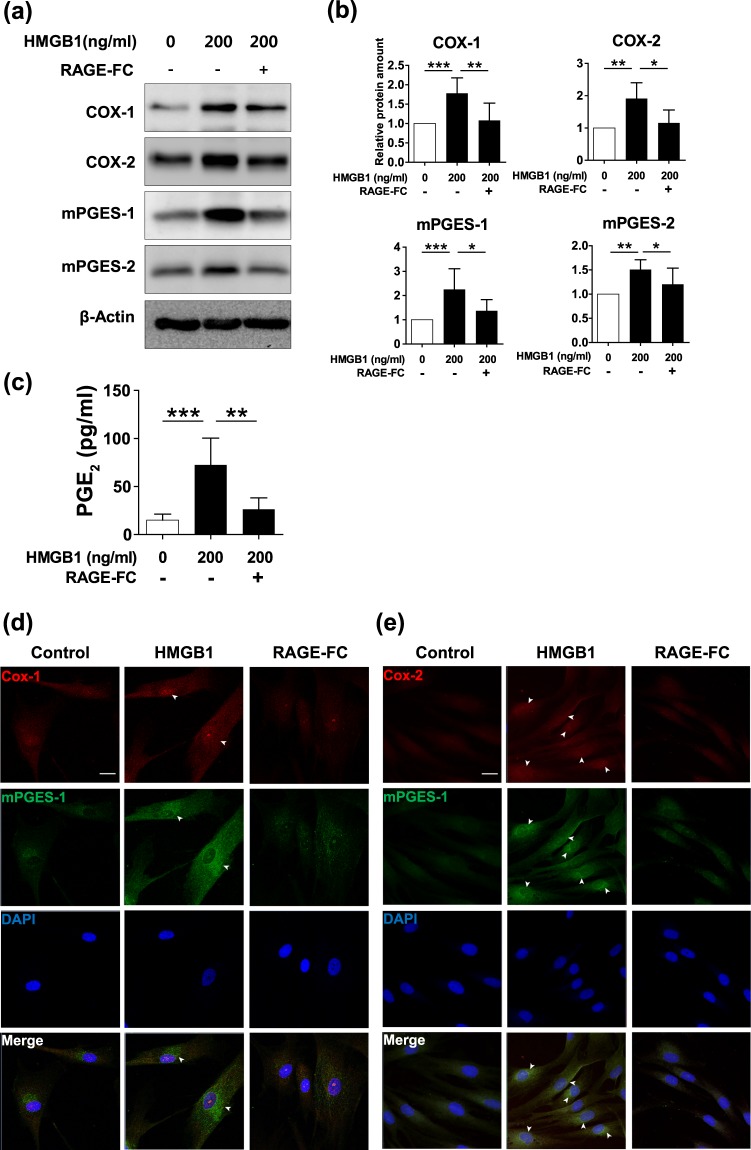
RAGE is the corresponding receptor for HMGB1 in hDPCs. Blocking RAGE (RAGE-FC) inhibited the effects of HMGB1 on prostaglandin metabolism. Cultured hDPCs were pre-treated with or without 10 μg/ml RAGE-FC for 30 min and then incubated with 200 ng/ml HMGB1 for 30 min. (**a**) The protein levels of COX-1, COX-2, mPGES-1, and mPGES-2 were measured using western blot analysis. (**b**) Protein bands were analysed using densitometry, and β-actin served as a loading control. The results are expressed as mean ± SD of three independent experiments. *p < 0.05, **p < 0.01, and ***p < 0.001 compared with control group using one-way ANOVA followed by Bonferroni’s test. (**c**) PGE_2_ production from cultured hDPCs was measured using ELISA. Cultured hDPCs were pre-treated with 10 μg/ml RAGE-FC for 30 min and then incubated with 200 ng/ml HMGB1 for 4 h. Data are representative of three independent experiments. The results are expressed as mean ± SD of three independent experiments. **p < 0.01 and ***p < 0.001 compared with control group using one-way ANOVA. Representative images of immunofluorescence staining for (**d**) COX-1 (red), (**e**) COX-2 (red), and mPGES-1 (green) in hDPCs pre-treated with 10 μg/ml RAGE-FC for 30 min and 200 ng/ml HMGB1 treatment for another 30 min. 4′-6-diamidino-2-phenylindole (DAPI; blue) was used to counterstain the nuclei. White arrowheads mark the expression of each enzyme in perinuclear region of hDPCs. Data are representative of three independent experiments. Scale bar = 20 μm.

Because HMGB1 binds to various target receptors, including TLR2, TLR4 and RAGE, we next wanted to determine the receptor pathway related to HMGB1-induced signalling in hDPCs. First, the effect of HMGB1 on RAGE was evaluated, which revealed that *RAGE* expression substantially increased in a time-dependent manner until 4 hours of HMGB1 treatment, and then subsequently decreased (Supplementary Fig. 5). Interestingly, treatment with a RAGE inhibitor, RAGE-FC, directly abrogated HMGB1-induced COX1, COX2, mPGES-1 and mPGES-2 expression in hDPCs (Fig. 4a,b). Specifically, while treatment with HMGB1 increased expression of these proteins by 1.5 to 2-fold, their expression returned to control levels when pre-treated with RAGE-FC (Fig. 4b). Similarly, the RAGE inhibitor suppressed the HMGB1-induced increase in PGE_2_ secretion from cultured hDPCs (Fig. 4c). These results were confirmed by immunofluorescent staining (Fig. 4d,e and Supplementary Fig. 6). However, the expressions of mPGES-1 and mPGES-2 were not altered by blocking antibodies against TLR2 and TLR4, other target receptors for disulfide-HMGB1 (Supplementary Fig. 7a). Instead, PGE_2_ production by reduced form was blocked by anti-TLR4 antibody but not by RAGE-FC (Supplementary Fig. 7b,c), which suggests modification in redox status of HMGB1 can affect its binding affinity to different target receptors. Collectively, these data suggest that HMBG1 increases PGE_2_ production in hair follicles via RAGE particularly for the disulfide-HMGB1.

### RAGE blockade inhibits HMGB1-induced hair shaft elongation in human hair follicles

**Figure 5:**
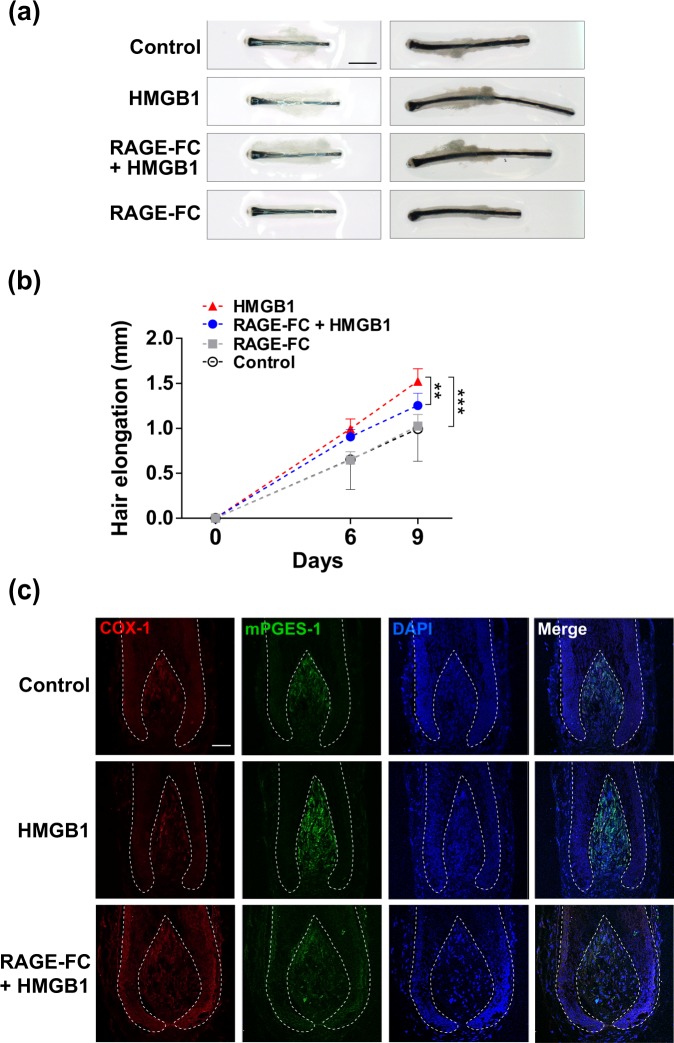
RAGE blockade inhibits HMGB1-induced hair shaft elongation in human hair follicles. Human hair follicles were pre-treated with 10 μg/ml RAGE-FC for 1 h followed by 200 ng/ml HMGB1 for 9 days. (**a**) Representative photos of hair follicles from each group are shown. (**b**) Quantification of hair shaft growth rates. The changes in hair shaft lengths were quantified at 6 and 9 days using a stereomicroscope. Data are shown as the mean ± SD of five independent donors (at least 120 follicles for each experiment. p-values were determined by ANOVA followed by Bonferroni test. **p < 0.01 and ***p < 0.001 compared with control group. (**c**) Human hair follicles were pre-treated with 10 μg/ml RAGE-FC for 1 h followed by 200 ng/ml HMGB1 for 3 days. Representative confocal images of hair follicles stained with COX-1 (red) and mPGES-1 (green) are shown. 4′-6-diamidino-2-phenylindole (DAPI; blue) was used to counterstain the nuclei. On day 3, each hair follicle was frozen and cryosectioned (6 μm). Data are representative of three independent experiments. Scale bar = 50 μm.

Finally, we wanted to evaluate the effect of RAGE inhibition on HMGB1-induced hair shaft elongation. To test this, hair follicles were pre-treated with RAGE-FC for 1 hour prior to HMGB1 treatment for 9 days, and then hair shafts elongation was measured. Importantly, hair follicles treated with HMGB1 and RAGE inhibitor were significantly shorter than those treated with HMGB1 alone (Fig. 5a,b). Immunofluorescent staining revealed that HMGB1 significantly increased COX-1 and mPGES-1 expression in the dermal papilla, while RAGE-FC pre-treatment inhibited this effect and returned their expression to control levels (Fig. 5c). These findings confirm that HMGB1 induces hair shaft elongation through RAGE signalling.

## Discussion

In this study, we selected HMGB1, a key DAMP, as a candidate mediator of trauma- or inflammation-induced hair growth. We confirmed that HMGB1 stimulated hair follicle elongation in an *ex vivo* hair organ culture and PGE_2_ secretion from hDPCs. Importantly, blocking RAGE inhibited HMGB1-induced expression of PGE_2_ synthases. These results suggest that HMGB1 induces PGE_2_ secretion from hDPCs through RAGE signalling, and that PGE_2_ stimulates the proliferation of neighbouring follicular matrix keratinocytes to enhance hair shaft elongation.

Prostaglandins regulate a variety of physiological activities such as inflammation, platelet aggregation, neurotransmitter release, and smooth muscle contraction^16^. One prostaglandin subset, prostaglandin D_2_, inhibits hair follicle elongation and promotes the onset of catagen, leading to the miniaturization of the hair follicle in androgenetic alopecia^13^. By contrast, PGE_2_ promotes hair growth in both mice and humans. Indeed, PGE_2_ treatment induces rapid hair growth after depilation, and subcutaneous or topical administration of 16,16-dimethyl PGE_2_ prevents radiation-induced alopecia in mice^17,18^. Furthermore, treatment with viprostol, a PGE_2_ analogue, increases hair growth in humans^19^. PGE_2_ also modulates cell proliferation and viability^20–22^. Thus, prostaglandins, particularly PGE_2_, are critically involved in hair growth.

PGE_2_ has also been previously associated with HMGB1. Specifically, HMGB1, in complex with IL-1β, induces mPGES-1 and PGE_2_ in synovial fibroblasts from patients with rheumatoid arthritis^23^. HMGB1, together with IL-1β, also activates *COX-2* and *mPGES-1*, significantly increasing the production of PGE_2_ in vascular smooth muscle cells^24^. As in previous studies, in our study, HMGB1 induced PGE_2_ production in hDPCs, a specialized subset of fibroblasts. This PGE_2_ production promoted hair shaft elongation. Importantly, DP express prostaglandin E receptors, which bind PGE_2_ ^25^, so HMGB1-induced PGE_2_ production could also promote hair growth by regulating DP activity in an autocrine and/or paracrine manner^22,25^.

In this study, the growth-promoting effects of HMGB1 were RAGE-dependent. RAGE binds many ligands, including HMGB1, and its expression has been found to be increased under acute or chronic inflammatory conditions^26^. RAGE signalling can also significantly increase PGE_2_ production from endothelial cells and synoviocytes^27,28^. Similarly, in our study, blocking RAGE inhibited PGE_2_ synthases, suggesting that PGE_2_ secretion from DPCs occurs through RAGE signalling. HMGB1, a RAGE ligand, is passively released from various cells during cellular necrosis and inflammation, but particularly from activated myeloid cells^9,29^. Rapidly released by damaged tissues, HMGB1 is an early stage mediator in trauma^30^. It also contributes to tissue repair by modulating chemotaxis, neoangiogenesis, and cell proliferation^31,32^. To our knowledge, our results are the first to show that HMGB1 also promotes hair growth.

Recently, growing evidences revealed that interaction between different redox forms of HMGB1 and their affinity of various target receptors is getting more complex^33–35^. From our observation, both forms of HMGB1, disulphide- and reduced-HMGB1, have potential capability to induce PGE_2_ from hDPCs but their primarily responding receptors can vary depending on redox status.

Counterintuitively, previous studies in alopecia areata have shown the contradictory effect of HMGB1 on hair follicle growth^36^. HMGB1 levels are significantly higher in the tissue and sera of these patients. But HMGB1 effect in alopecia areata results from its immunostimulating action, which eventually induces the apoptosis of the hair follicle keratinocyte driven by autoimmune response^37^. And other factors including concentration of HMGB1 in the local milieu can determine the consequence. For example, the migration and chemotaxis of polymorphonuclear neutrophils is inhibited at low HMBG1 concentrations, but enhanced at high concentrations^38^. Therefore, future studies are needed to understand such double-sided biological effect of HMGB1 in a complicated environment such as inflamed hair follicles. In summary, our results demonstrated that HMGB1, an inflammatory mediator, markedly stimulated hair shaft elongation in both *in vitro* and *ex vivo* models by stimulating PGE_2_ production. This effect was dependent on RAGE, and blocking this receptor attenuated the effects of HMGB1 on hair growth. Thus, HMGB1 signalling may explain the paradoxical hypertrichosis that occurs following laser epilation and hypertrichosis in other inflammatory or traumatic circumstances. Ultimately, our study suggests that HMGB1 may be a novel therapeutic target for hair growth and alopecia treatment.

## Methods

### Hair follicle sources and isolation of dermal papilla cells

This study was approved by the Institutional Review Board at Severance Hospital, and all sample donors provided written informed consent. All experimental procedures using human materials were conducted according to the Declaration of Helsinki Principles.

Occiput scalp skin containing mainly anagen VI hair follicles was obtained from disposed excess skin samples derived from patients undergoing elective plastic surgery. Hair follicles were isolated under a stereo dissecting microscope, and dermal papilla were separated from individually isolated hair follicles as previously described^39^. Hair follicles that were morphologically considered to be in anagen were used in this study.

### Cultivation of isolated dermal papilla cells

Dermal papilla cells (hDPCs) were cultured in Dulbecco’s modified Eagle’s medium (DMEM; Hyclone, Logan, UT, USA) supplemented with 10% fetal bovine serum (FBS; Hyclone) and antibiotic/antimycotic solution (Gibco BRL, Gaithersburg, MD, USA) containing penicillin and streptomycin. Cells were incubated at 37 °C in a 5% CO_2_ incubator. All cultures used for experiments were in the third or fourth passage. Depending on the experiment, the media were supplemented with different concentrations of recombinant human HMGB1 (HMGB1; R&D systems, Minneapolis, MN, USA) or HMGB1 with antibodies blocking receptor for advanced glycation end-products (RAGE-FC, R&D systems), anti-TLR2 (BioLegend, San Diego, CA, USA), or anti-TLR4 (eBiosicence, San Diago, CA, USA) neutralizing antibodies. For the analysis on the effects of different forms of HMGB1, 200 ng/ml of A-box and 200 ng/ml of B-box (HMGBiotech, Milano, Italy), and 5 mM of dithiothreitol (DTT; Sigma–Aldrich, St. Louis, MO, USA) were used.

### Hair follicle organ culture

Normal human scalp skin hair follicles in the anagen VI stage of the hair cycle^40^ were isolated as previously described^41^ with slight modifications. Briefly, after separation of the hair follicles under a binocular dissecting microscope, the proximal two-thirds of anagen hair follicles located in the subcutaneous fat were isolated using watchmakers forceps, and subsequently collected in a 35-mm culture dish containing complete hair follicle culture medium (Williams E; Gibco BRL) supplemented with 2 mM/L-glutamine (Gibco BRL), 10 ng/ml hydrocortisone (Sigma-Aldrich, St Louis, MI, USA), and 10 μg/ml insulin (Invitrogen, Carlsbad, CA, USA). Three isolated hair follicles were then cultured for 9 days in each individual wells of a 24-well plate containing 500 µL of complete hair follicle culture medium. Depending on the experiment, the media were supplemented with different concentrations of recombinant human HMGB1 (R&D systems) or HMGB1 with antibodies blocking receptor for advanced glycation end-products (RAGE-FC, R&D systems). A positive control, minoxidil (Sigma–Aldrich, St. Louis, MO, USA) was used in a concentration of 1 μM. The culture medium was replaced every two days. Each experiment was repeated with hair follicles harvested from different donors. Every third day, photographs of the hair shafts were taken, and the length of each hair follicle was measured. The hair cycle stage of each follicle was assessed and classified by morphological criteria^40,42^.

### Methyl thiazolyl-diphenyl-tetrazolium bromide (MTT) cell viability assay

The influence of HMGB1 on hDPC proliferation was assessed by MTT cell proliferation assay^43^. hDPCs were seeded at a density of 1.6 × 10^3^ cells/well on 96-well plates. After 18 h of incubation, the cells were washed with PBS and cultured for 24 h within 200 μl of FBS-free DMEM, together with the indicated concentrations of HMGB1. Then, the medium was removed and 100 μl of 5 mg/ml MTT [3-(4,5-dimethylthiazol-2-yl)-2,5-diphenyl-2H-tetrazolium bromide] (Sigma–Aldrich, St. Louis, MO, USA) in PBS was added. At the end of 4 h incubation, the plates were centrifuged, and the untransformed MTT was removed. After 100 μl of dimethyl sulfoxide (DMSO; Sigma-Aldrich, St. Louis, MI, USA) was added to each well, the plates were shaken for 10 min. The optical density at 570 nm was determined using an ELISA reader. The cell viability rates were calculated from the optical density (OD) readings and are represented as percentages of the control value (untreated cells).

### Enzyme-linked immunosorbent assay (ELISA) for PGE_2_

Prostaglandin E2 concentrations in the medium from cultured hDPCs were measured by enzyme immunoassay (EIA kit; Cayman Chemicals, Ann Arbor, MI, USA) according to the manufacturer’s instructions. Briefly, the hDPCs were cultured in triplicates at 7.5 × 10^4^ cells/ml onto 48-well plates. After 18–24 h of incubation, the cells were washed with PBS and cultured in serum-free condition for 24 h. hDPCs were incubated with HMGB1 at different time points (0, 0.5, 1, 2, and 4 h) or stimulated with 200 ng/ml HMGB1 for 4 h followed by RAGE-FC for 30 min, respectively. Then, 50 µl of cultured medium was incubated in goat anti-mouse IgG-coated plates with a PGE_2_-acetylcholinesterase conjugate (PGE_2_ tracer) and PGE_2_ monoclonal antibody overnight at 4 °C. Finally, 200 µl of Ellman’s reagent was added to each well for 90 min at room temperature and the plate was read at 412 nm.

### Human cytokine array analysis

Supernatant of hDPCs cultured with or without 200 ng/ml HMGB1 was collected at 48 h. Levels of multiple cytokines were assayed using the Human Growth Factor Antibody Array (Abcam, Cambridge, UK) following the manufacturer’s instructions. Briefly, array membranes immobilized with capture antibodies were incubated with 1 ml of the supernatant for 1 h at room temperature. Then, a biotin-conjugated detection antibody cocktail was added to the membranes for overnight at 4 °C. The membranes were then incubated with horseradish peroxidase-conjugated streptavidin, and signal was detected using an ECL Plus kit (Millipore Corporation, Billerica, MA, USA). Images of each array were captured by LAS-4000 (GE Healthcare, Pittsburgh, PA, USA). The signal intensity was calculated using Image J (NIH, Bethesda, MD, USA).

### Protein extraction and western blot

Protein expression was assessed with western blots. Briefly, collected cells were lysed with RIPA lysis buffer (GenDEPOT, Houston, TX, USA), and protein concentration was quantified using the BCA assay. Protein samples were then resolved by SDS-PAGE and transferred onto a nitrocellulose membrane (GE Healthcare). After blocking, the membranes were incubated with the respective primary mouse antibodies: anti-cyclooxygenase-1 (COX-1; 1:1000), anti-cyclooxygenase-2 (COX-2; 1:500; Abcam, Cambridge, UK), anti-microsomal PGE synthase-1 (mPGES-1; 1:1000), anti-microsomal PGE synthase-2 (mPGES-2; 1:500), anti- cytosolic PGE synthase (cPGES; 1:200; Cayman Chemicals), or anti-β-actin (1:1000; Cell Signalling Technology, Beverly, MA, USA). The membranes were then incubated with peroxidase-conjugated affinity-purified goat anti-rabbit IgG or goat anti-mouse IgG secondary antibody (1:10000; GenDEPOT), and the protein bands were detected using an ECL Plus kit (Millipore Corporation, Billerica, MA, USA).

### RNA isolation and quantitative real-time PCR

Total RNA from cells was isolated with Trizol reagent (Qiagen GmbH, Hilden, Germany). For real-time PCR (RT-PCR), purified RNA was oligo(dT)-primed for first-strand cDNA synthesis (Superscript III kit; Invitrogen). A quantitative SYBR Green RT-PCR kit (Applied Biosystems, Warrington, UK) was used with a Step One Plus RT-PCR System (Applied Biosystems). Quantitative RT-PCR was performed using the following sequence-specific primers: GAPDH (forward: 5′-TGGAAATCCCATCACCATCTTC-3′ and reverse: 5′-CGCCCCACTTGATTTTGG-3′), COX-1 (forward: 5′-TGCGCTCCAACCTTATCCC-3′ and reverse: 5′-AGAGGGCAGAATACGAGTGTAA-3′), COX-2 (forward: 5′-CTGGCGCTCAGCCATACAG-3′ and reverse: 5′-CGCACTTATACTGGTCAAATCCC-3′), mPGES-1 (forward:5′-GAAGAAGGCCTTTGCCAACC-3′ and reverse: 5′-ATGGTCTCCATGTCGTTCCG-3′), mPGES-2 (forward:5′-TACCAGGTGGTGGAGGTGAA-3′ and reverse: 5′-TGCGAGCTTTCTCCTTCCTG-3′), cPGES (forward: 5′-TGGCTTAGTGTCGACTTCAAT-3′ and reverse: 5′-TCCTCATCACCACCCATGTTG-3′), aldo-keto reductase family 1 member C1 (AKR1C1) (forward: 5′-TTGCATGAGGTCTGCCA-3′ and reverse: 5′-GCTGTAGCTTGCTGAAAT-3′), aldo-keto reductase family 1 member C3 (AKR1C3) (forward: 5′-GCCTGTATTGGGATTTGGCAC-3′ and reverse: 5′-TCTATATGGCGGAACCCAGC-3′), carbonyl reductase 1 (CBR1) (forward: 5′-AAGATTGGCGTCACCGTTCT-3′ and reverse: 5′-GTAAATGCCCTTTGGACCAACT-3′), prostaglandin-H2 D-isomerase (PTGDS) (forward: 5′-AACCAGTGTGAGACCCGAAC-3′ and reverse: 5′-CTGACACGGAGTAGGTGCTG-3′), alkaline phosphatase (ALPL) (forward: 5′-TCACTCTCCGAGATGGTGGT-3′ and reverse: 5′-TTTCCTTCATGGTGCCCGT-3′), and vascular endothelial growth factor (VEGF) (forward: 5′-CTGCTCTACCTCCACCATGC-3′ and revers: 5′-AGCTGCGCTGATAGACATCC-3′). The PCR was conducted under the following conditions: Denaturation at 95 °C for 15 sec, 40 amplification cycles of annealing at 60 °C for 30 sec, and extension at 72 °C for 30 sec. All samples were run in triplicate, and relative gene expression was determined using the 2^−ΔΔCt^ method, with *GAPDH* as the normalization standard.

### Immunofluorescence

Immunofluorescence staining was performed on hDPCs and 5-μm frozen sections of hair follicles. The hDPCs were plated in 8 chamber slides (LAB-TEK, Rochester, NY, USA) at a density of 1.6 × 10^3^ cells per well and cultured in serum-free DMEM with 200 ng/ml HMGB1 or with blocking antibodies.

Hair follicles and hDPCs were fixed in 4% paraformaldehyde for 15 min, and then permeabilized with 0.1% Triton X-100 (Sigma-Aldrich) and blocked with 10% goat serum. The following primary antibodies were used: anti-Ki-67 (DAKO, Carpinteria, CA, USA), mouse anti-COX-1 (1:100; Abcam, Cambridge, MA, USA), FITC-conjugated anti-COX-2 (1:100; Abcam), rabbit anti-mPGES-1 (1:100; Cayman Chemicals) and rabbit anti-mPGES-2 (1:100; Cayman Chemicals). After washing with PBS, samples were incubated with the appropriate secondary antibodies: Alexa Fluor 488 goat anti-rabbit IgG, Alexa Fluor 635 goat anti-mouse IgG (Life Technologies, Eugene, OR, USA) and Alexa Fluor 635 goat anti-rabbit (Abcam). Slides were washed in PBS and counterstained with 4,6-diamidino-2-phenylindole (DAPI) (Life Technologies). Immunofluorescence images were acquired using Zeiss LSM 700 confocal laser scanning microscopy software (Carl Zeiss, Oberkochen, Germany). For quantitative analyses, Ki-67-positive cells were counted and normalized to the number of DAPI-stained cells using ZEN 2009 software (Carl Zeiss).

### Statistical analysis

Statistical analyses were conducted using Graph Pad Prism, version 4.03 (GraphPad Software, Inc., San Diego, CA, USA). Data are expressed as mean ± SD for parametric data, and as median and interquartile range for nonparametric data. The significance of differences between two groups was determined using a two-tailed Student’s t-test or Wilcoxon signed ranks test. For multiple comparisons, a one-way ANOVA with a Bonferroni test was used. A P-value < 0.05 was considered statistically significant. Significance is indicated in the figures as follows: *P < 0.05; **P < 0.01; ***P < 0.001.

## Supplementary information

Supplementary Figure S1-S7
