# Supplementary material for: HMGB1 promotes hair growth via the modulation of prostaglandin metabolism
Source: Sci Rep. 2019 Apr 30;9:6660. doi: 10.1038/s41598-019-43242-2 (PMC6491442; doi:10.1038/s41598-019-43242-2)
Supplement: Supplementary file 1 — Supplementary Figure S1-S7 [file 41598_2019_43242_MOESM1_ESM.pdf]

SUPPLEMENTARY DATA

HMGB1 promotes hair growth via the modulation of prostaglandin metabolism

Ji-Hye Hwang[1,3], Howard Chu[1,3], Yuri Ahn[1], Jino Kim[2], Do-Young Kim[1*]

Contents

Figure S1. **HMGB1 enhanced the proliferation of cultured hDPCs as determined by MTT assay.**

Figure S2. Cytokine array of supernatant of cultured media from hDPCs treated with HMGB1.

Figure S3. HMGB1 increases PGE_2 production in hDPCs in a time-dependent manner.

Figure S4. Both redox forms of HMGB1 induce PGE_2 production but not by isolated box constructs, A-box or B-box.

Figure S5. HMGB1 regulates RAGE mRNA levels in hDPCs in a time-dependent manner.

Figure S6. Blockade of RAGE reduces the expression of mPGES-2 in HMGB1-treated hDPCs.

Figure S7. Disulfide HMGB1-induced PGE_2 secretion is dependent on RAGE in hDPCs.

SUPPORTING INFORMATION

Figure S1. **HMGB1 enhanced the proliferation of cultured hDPCs as determined by MTT assay.** hDPCs were treated with various concentrations (25, 50, 100, and 200 ng/ml) of HMGB1 for 24 h. Assays were repeated in triplicate. Multiple comparisons were performed by one-way ANOVA followed by Bonferroni's test. Values are means ± SD. *p < 0.05 and ***p < 0.001 compared with the vehicle-treated control.

Figure S2. Cytokine array of supernatant of cultured media from hDPCs treated with HMGB1. Cytokine production was screened using proteome profiler arrays. hDPCs were treated with 200 ng/ml HMGB1 for 48 h. Supernatants were harvested and used for experiments. Data were mean ± SD from two independent experiments. Data were normalized to the intensity of positive control (Pos, three pairs of dots at the corners).

Figure S3. HMGB1 increases PGE_2 production in hDPCs in a time-dependent manner.

hDPCs were treated with 200 ng/ml HMGB1. The concentration of PGE_2 in culture supernatants collected at different time points (1, 2, 4, and 24 h) were analysed by ELISA. Data are representative of three independent experiments. The results are expressed as mean ± SD of three independent experiments. **p < 0.01 compared with control group using one-way ANOVA.

Figure S4. Both redox forms of HMGB1 induce PGE_2 production but not by isolated box constructs, A-box or B-box.

(a) hDPCs were incubated with 200 ng/ml HMGB1 (R&D), A-box (HMGBiotech, Milano, Italy), or B-box (HMGBiotech, Milano, Italy) for 4 h. The concentration of PGE_2 in culture supernatants were measured by ELISA. Data are representative of three independent experiments. The results are expressed as mean ± SD of three independent experiments. **p < 0.01 compared with control group using one-way ANOVA. (b) Electrophoretic pattern of different HMGB1 redox forms by Western

blotting. HMGB1 in the presence or absence of 5 mM dithiothreitol (DTT) for 1 h. (c) The concentration of PGE_2 in culture supernatants was measured using ELISA. hDPCs were incubated with (reduced HMGB1) or without 5 mM of DTT (disulfide HMGB1). The results are expressed as mean ± SD of three independent experiments. **p < 0.01; n.s., not significant compared with control group using one-way ANOVA.

Figure S5. HMGB1 regulates RAGE mRNA levels in hDPCs in a time-dependent manner.
hDPCs were cultured with 200 ng/ml HMGB1, and *RAGE* expression was quantified by real-time PCR. The relative mRNA expression was normalized to *GAPDH*. The results are expressed as mean ± SD of three independent experiments. *p < 0.05 and ***p < 0.001 compared with control group using one-way ANOVA followed by Bonferroni's test.

Figure S6. Blockade of RAGE reduces the expression of mPGES-2 in HMGB1-treated hDPCs.
Immunofluorescence staining for COX-1 (red), COX (green), and mPGES-2 (yellow) in hDPCs pre-treated with 10 μg/ml RAGE-FC for 30 min and 200 ng/ml HMGB1 treatment for another 30 min. 4'-6-diamidino-2-phenylindole (DAPI; blue) was used to counterstain the nuclei. White arrowheads mark the expression of PGE_2 synthases (COX-1, COX-2, or mPEGS-1) in perinuclear region of hDPCs. Data are representatives of three independent experiments. Scale bar = 20 μm.

Figure S7. Disulfide HMGB1-induced PGE_2 secretion is dependent on RAGE in hDPCs.

The hDPCs were pre-incubated with blocking antibodies (10 μg/ml RAGE-FC, 10 μg /ml anti-TLR2, and 10 μg/ml anti-TLR4) for 30 min and incubated with 200 ng/ml HMGB1 for 30 min (a) or 4 h (b-c). (a) The expression of mPGES-1, mPGES-2 was determined by western blot. (b-c) hDPCs were stimulated with HMGB1 prepared in presence or absence of DTT after pre-treatment with the blocking antibodies and the supernatants were examined for levels of PGE_2 by ELISA. Data are shown are mean ± SD. **p < 0.01; ***p < 0.001; n.s., not significant in comparison using one-way ANOVA.

Figure S1

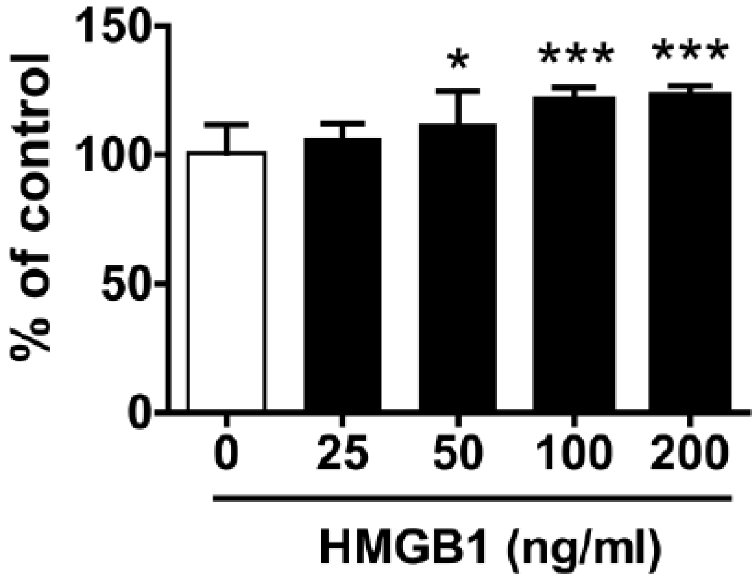

Figure S2

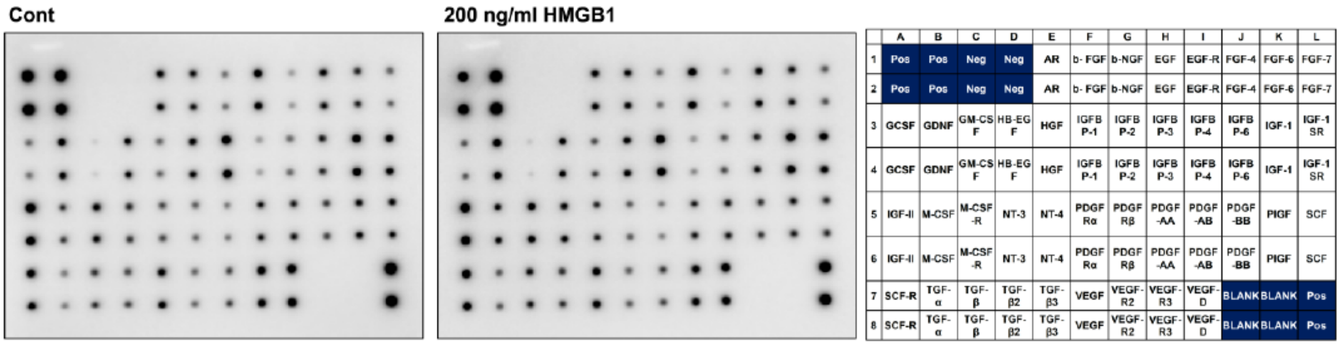

	A	B	C	D	E	F	G	H	I	J	K	L
1	Pos	Pos	Neg	Neg	AR	b- FGF	b-NGF	EGF	EGF-R	FGF-4	FGF-6	FGF-7
2	Pos	Pos	Neg	Neg	AR	b- FGF	b-NGF	EGF	EGF-R	FGF-4	FGF-6	FGF-7
3	GCSF	GDNF	GM-CSF	HB-EGF	HGF	IGFBP-1	IGFBP-2	IGFBP-3	IGFBP-4	IGFBP-6	IGF-1	IGF-1 SR
4	GCSF	GDNF	GM-CSF	HB-EGF	HGF	IGFBP-1	IGFBP-2	IGFBP-3	IGFBP-4	IGFBP-6	IGF-1	IGF-1 SR
5	IGF-II	M-CSF	M-CSF-R	NT-3	NT-4	PDGF Rα	PDGF Rβ	PDGF-AA	PDGF-AB	PDGF-BB	PIGF	SCF
6	IGF-II	M-CSF	M-CSF-R	NT-3	NT-4	PDGF Rα	PDGF Rβ	PDGF-AA	PDGF-AB	PDGF-BB	PIGF	SCF
7	SCF-R	TGF-α	TGF-β	TGF-β2	TGF-β3	VEGF	VEGF-R2	VEGF-R3	VEGF-D	BLANK	BLANK	Pos
8	SCF-R	TGF-α	TGF-β	TGF-β2	TGF-β3	VEGF	VEGF-R2	VEGF-R3	VEGF-D	BLANK	BLANK	Pos

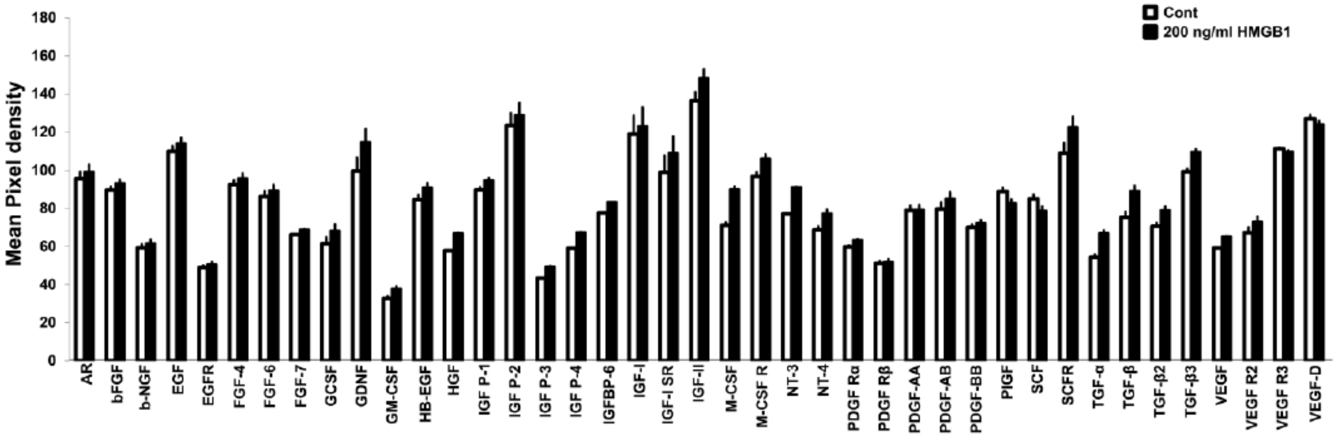

Figure S3

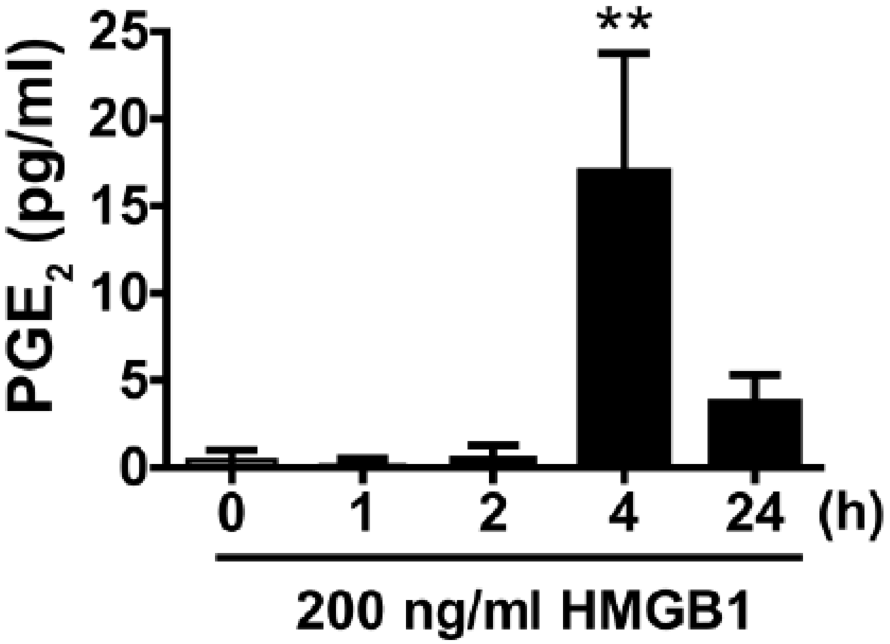

Figure S4

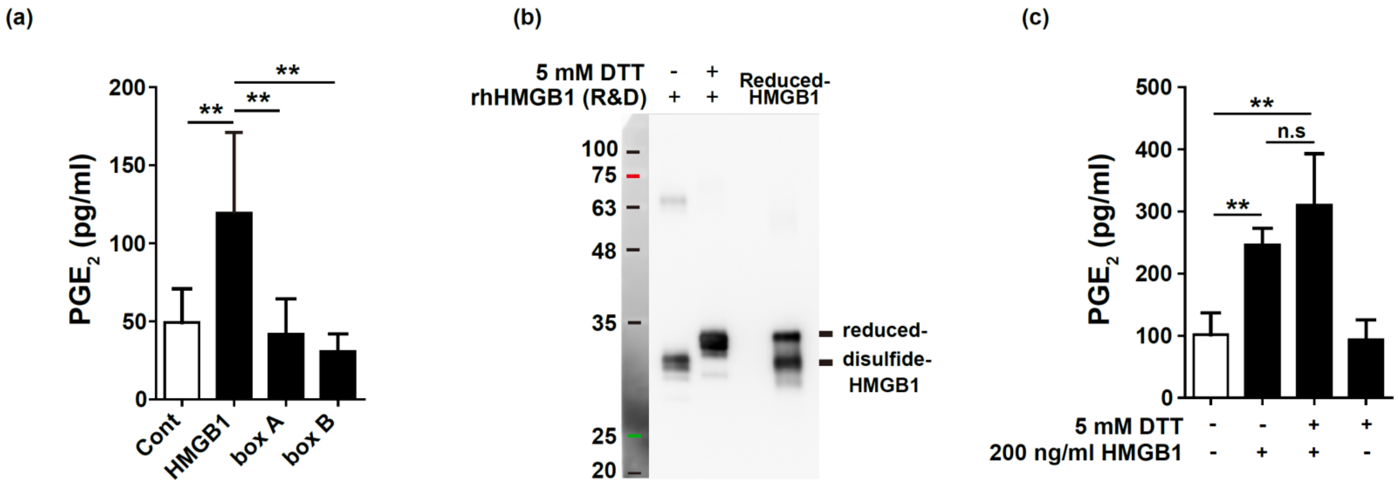
(a)
PGE2 (pg/ml)
200
150
100
50
0
**
**
**
Cont
HMGB1
box A
box B
(b)
5 mM DTT - +
rhHMGB1 (R&D) + +
Reduced-HMGB1
100
75
63
48
35
25
20
reduced-
disulfide-
HMGB1
(c)
PGE2 (pg/ml)
500
400
300
200
100
0
**
n.s
**
5 mM DTT - - + +
200 ng/ml HMGB1 - + + -

Figure S5

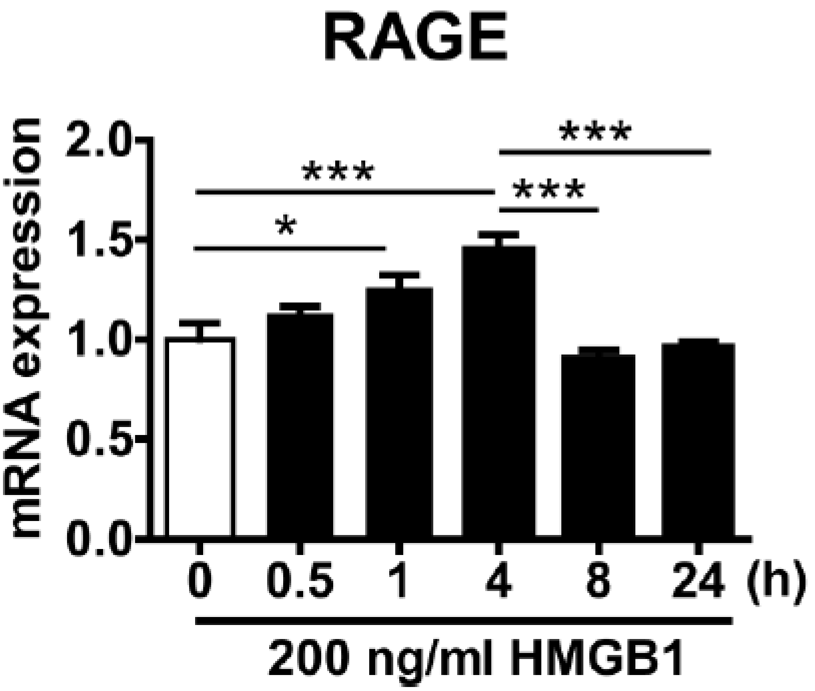

Figure S6

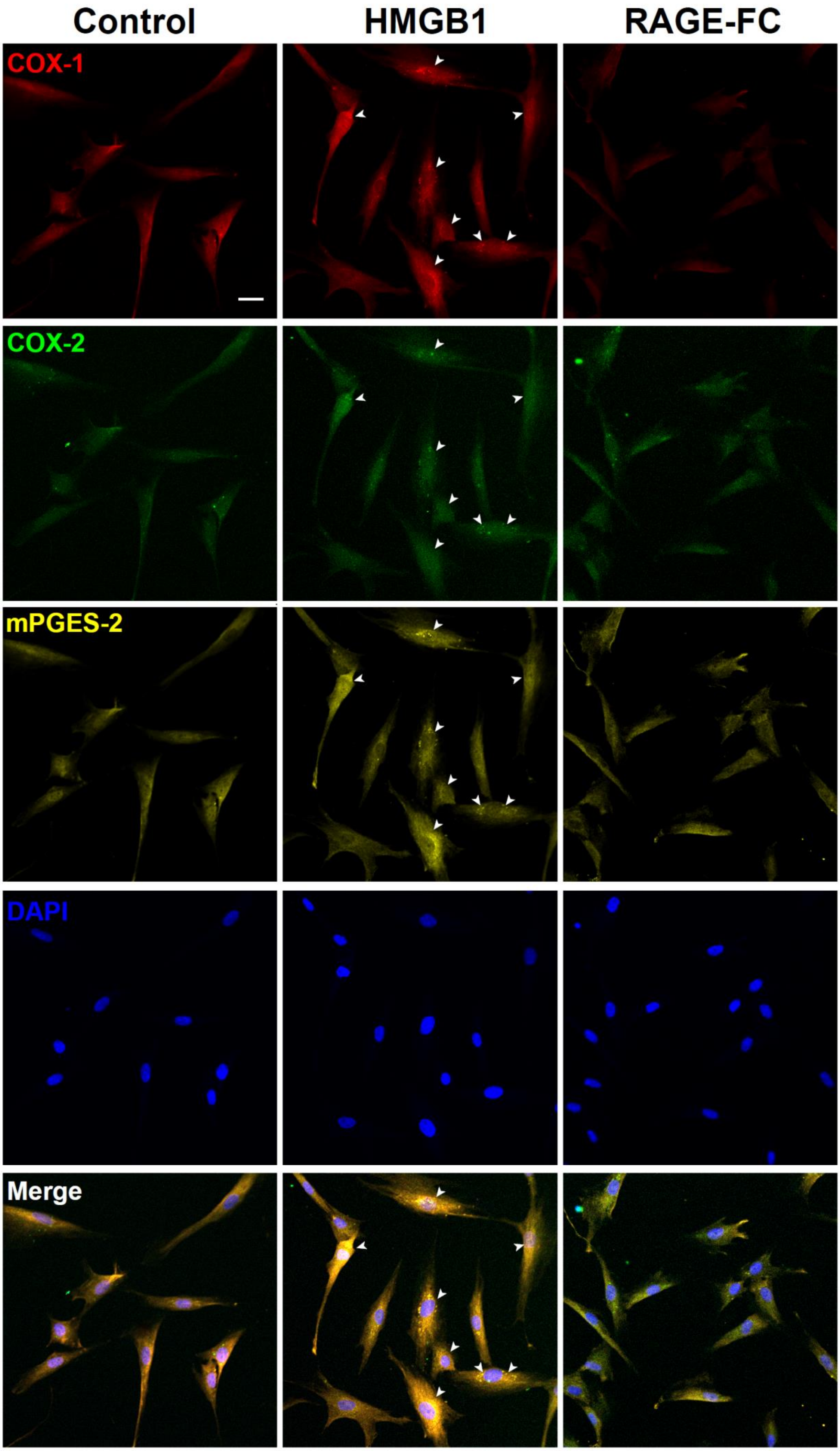

Figure S7

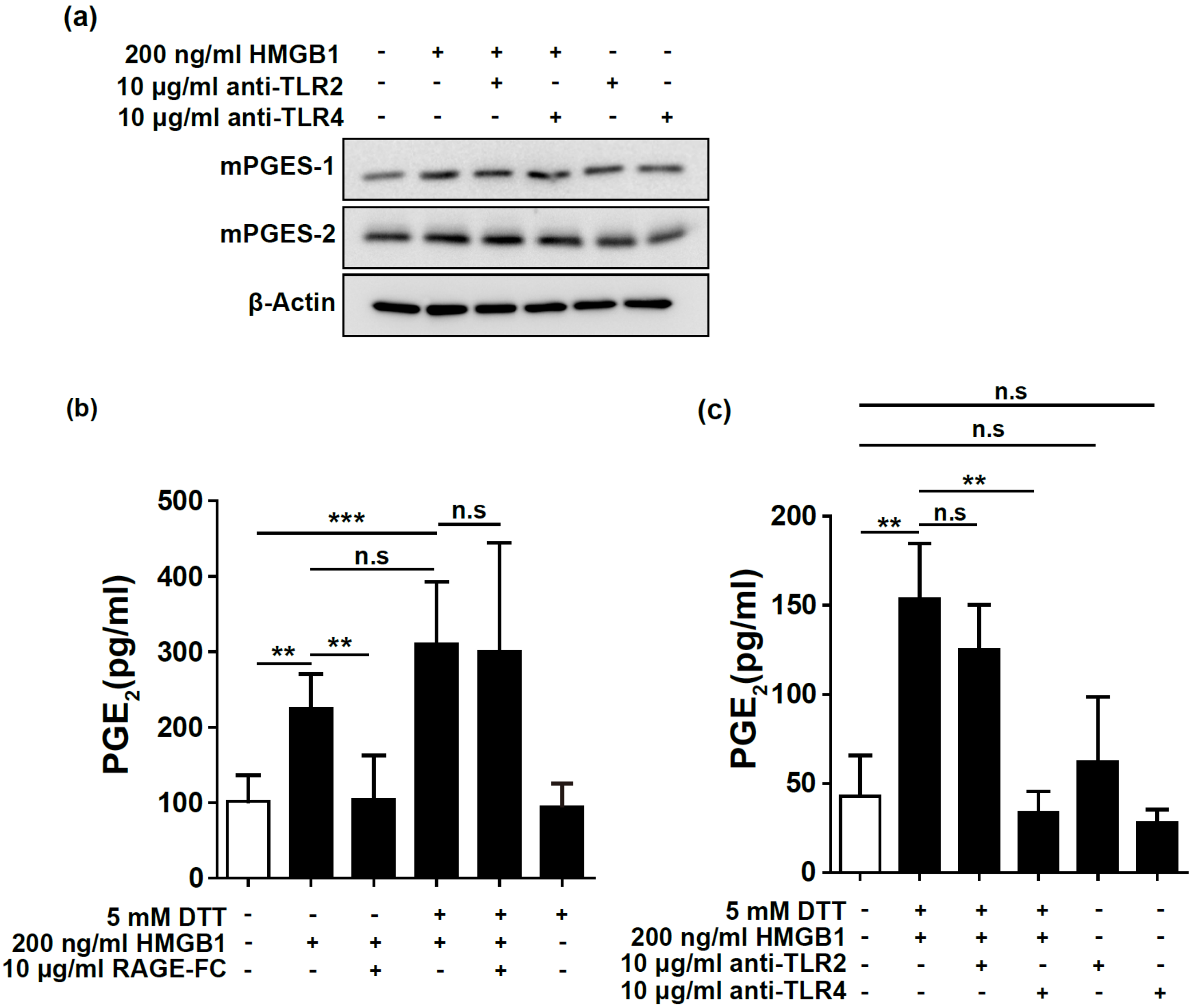
(a)
200 ng/ml HMGB1 - + + + - -
10 μg/ml anti-TLR2 - - + - + -
10 μg/ml anti-TLR4 - - - + - +
mPGES-1
mPGES-2
β-Actin
(b)
PGE2(pg/ml)
500
400
300
200
100
0
***
n.s
n.s
**
**
5 mM DTT - - - + + +
200 ng/ml HMGB1 - + + + + -
10 μg/ml RAGE-FC - - + - + -
(c)
n.s
n.s
**
**
n.s
PGE2(pg/ml)
200
150
100
50
0
5 mM DTT - + + + - -
200 ng/ml HMGB1 - + + + - -
10 μg/ml anti-TLR2 - - + - + -
10 μg/ml anti-TLR4 - - - + - +
